# Catalytically-active porous assembly with dynamic pulsating motion for efficient exchange of products and reagents

**DOI:** 10.1038/s42004-020-0259-4

**Published:** 2020-01-24

**Authors:** Shanshan Wu, Liping Huang, Yu Hou, Xin Liu, Jehan Kim, Yongri Liang, Jiong Zhao, Liwei Zhang, Hongbing Ji, Myongsoo Lee, Zhegang Huang

**Affiliations:** 1 Fine Chemical Industry Research Institute and PCFM Lab, School of Chemistry, Sun Yat-sen University, Guangzhou, 510275 PR China; 2 State Key Laboratory for Supramolecular Structure and Materials, College of Chemistry, Jilin University, Changchun, 130012 PR China; 3 Pohang Accelerator Laboratory, Postech, Pohang, Gyeongbuk Korea; 4 College of Materials Science and Engineering, Beijing Institute of Petrochemical Technology, Beijing, 102617 PR China; 5 Department of Applied Physics, The Hong Kong Polytechnic University, Hung Hom, Kowloon, Hong Kong

**Keywords:** Porous materials, Self-assembly

## Abstract

Despite recent advances in the use of porous materials as efficient heterogeneous catalysts which operate through effectively trapping reagents in a well-defined space, continuously uptaking reagents to substitute products in the cavity for efficient product turnover still remains challenging. Here, a porous catalyst is endowed with ‘breathing’ characteristics by thermal stimulus, which can enable the efficient exchange of reagents and products through reversible stacking from inflated aromatic hexamers to contracted trimeric macrocycles. The contracted super-hydrophobic tubular interior with pyridine environment exhibits catalytic activity towards a nucleophilic aromatic substitution reaction by promoting interactions between concentrated reagents and active sites. Subsequent expansion facilitates the exchange of products and reagents, which ensures the next reaction. The strategy of mesoporous modification with inflatable transition may provide a new insight for construction of dynamic catalysts.

## Abstract (web summary)

Porous materials are efficient heterogeneous catalysts, but the continuous uptake of reagents to substitute products in the cavities remains unsettled. Here the authors fabricate a self-assembled tubular structure with breathing characteristics for efficient exchange of reactants and products in a nucleophilic substitution reaction.

## Introduction

In nature, enzymatic reactions modulated by various effects with continuously trapping reactants and releasing products in well-defined spaces, such as photosynthesis, are of great significance to achieve vital movements in living organism^1–6^. Inspired by these systems, artificial catalysts with specific apertures have been developed to promote proximity effects by increasing the local concentration of substrates^7–10^. Additionally, their catalytic activity can be controlled by altering the environment around active centers in response to external triggers^11–16^. However, continuous uptake of reagents to exchange products in the confined active space for efficient product turnover still remains greatly challenging. Most porous structures are based on rigid and robust pores, where the products occupy the whole cavity and thus obstruct subsequent effective collisions. Therefore, artificial catalysts that enable dynamic uptake of reagents to substitute products in hollow aperture by external triggers for vital reaction are highly desirable.

Spontaneous assembly of small molecular modules driven by noncovalent interactions is key to creating stimuli-responsive dynamic devices. The expansion of transient states facilitates exchange of objects due to enhanced entropy in the aperture while the subsequent contraction accelerates effective collision between reagents^17,18^. Nevertheless, these transient pores based on noncovalent interaction are too delicate to ensure effective exchange. Among these self-assembled systems, aromatic rod amphiphiles composed of conjugated carbon and hydrophilic dendritic segments can easily aggregate into porous structures with hydrophobic characteristics, which are well suitable as defined scaffolds for organic catalysis^19–23^. Compared with traditional rigid porous structures, the hydrophobic pores based on di-block molecular assembly possess remarkable recognition for organic objects. For example, the hollow spherical adsorbents from folded aromatic rod assembly exhibited excellent removal efficiency for organic contaminants from waste water^24^. Well-defined helical polymers could recognize the size and chirality of higher fullerenes and selectively extract the enantiomers^25^. Introduction of an active atom such as nitrogen or metal into conjugated carbon substrates would serve as active sites for effective collision within aromatic apertures^26–29^. In contrast to traditional rigid porous structures, supramolecular catalyst was able to spontaneously release products through disassembly and recover catalytic performance by reassembly^30^.

**Fig. 1:**
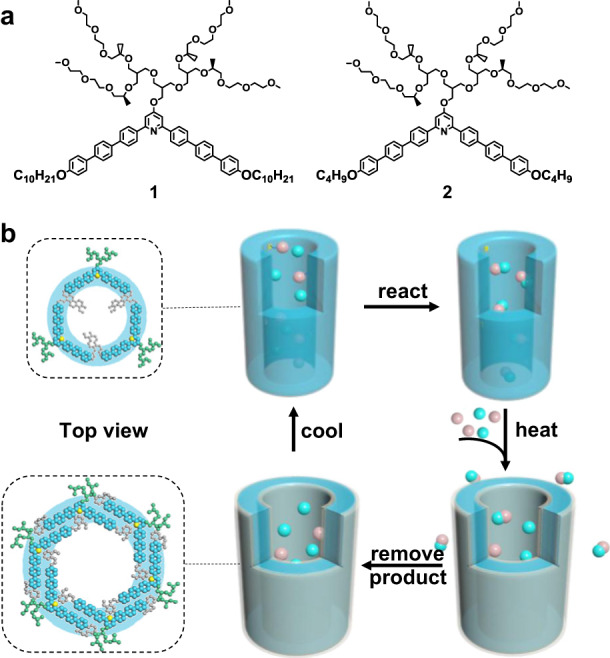
Molecular structure and thermo-responsive tubular catalyst. **a** Molecular structures of **1** and **2**. **b** Schematic representation of thermo-responsive tubular catalyst with inflated transition. Cyan and pink balls stand for R1 and R2 while the combination of cyan and pink balls represents product.

As hinted from recognition and regeneration of supramolecular apertures based on tunable assembly, herein we report a tubular catalyst with reversible contraction−expansion assembly for efficient exchange of products and reagents. This “pulsating” transition of the tubules is achieved by the introduction of thermo-responsive alkyl block into pyridine-based aromatic amphiphiles. The walls of the tubules are fabricated through self-stacking of trimeric bent-shaped segments that are capable of readily inflating into hexameric macrocycles by thermo triggers (Fig. 1). Due to the periodical distribution of nitrogen on the internal wall surfaces in the trimeric state, the contracted tubules can act as catalysts for a proof-of-principle nucleophilic aromatic substitution (S_N_Ar) reaction. Nonetheless, the hexameric transition expanded from trimeric macrocycles blocks the active sites, which can stop the reaction immediately. Importantly, the expansion of aromatic pores with specific recognition promotes the exchange of product and reagents to ensure continuous product turnover. The regulable modification of porous tubules for efficient exchange may provide a new insight for construction of dynamic catalyst.

## Results and discussion

### The design of catalyst

**Fig. 2:**
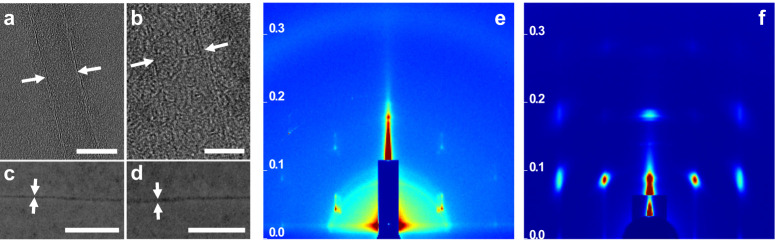
Structural characterization of tubules 1 and 2. TEM micrographs of **1** (**a**) and **2** (**b**) from aqueous solution (0.01 wt%), scale bar is 5 nm. STEM images of **1** (**c**) and **2** (**d**) from aqueous solution (0.01 wt%), scale bar is 50 nm. 2D XRD patterns of **1** (**e**) and **2** (**f**) from aqueous solution (0.02 wt%), *y-*axis: *q* space [Å^−1^]. For details, see Supplementary Fig. 4.

The tubular devices were derived from dynamic assembly of bent-shaped aromatic amphiphiles containing an active atom of nitrogen at the inner position with thermo-responsive alkyl chains at both ends, which were decorated by hydrophilic oligoether dendron at its apex (Fig. 1a, Supplementary Fig. 1). To verify the influence brought by alkyl chains, the assemblies of **1** and **2** with decyl and butyl terminal blocks were observed in aqueous solution. As confirmed by transmission electron microscopy (TEM) from 0.01 wt% aqueous solution, both samples showed two parallel dark lines with a uniform distance of 4.2 nm for **1** and 6.5 nm for **2**, indicative of tubular formation with defined walls (Fig. 2a, b). To further understand the porous tubular structures, scanning transmission electron microscopy (STEM) was performed with a probe aberration corrector and internal diameter was successfully obtained as 2.5 nm for **1** and 4.1 nm for **2**, respectively (Fig. 2c, d). The observed inner and outer diameter of tubular wall indicated that the elongation of alkyl chains induced the tubular contraction on the contrary. To explain the intriguing consequences, two-dimensional X-ray diffraction (2D XRD) was additionally carried out with thin membranes. **1** displayed a hexagonally ordered pattern in small-angle region with the correspondence lattice of 6.0 nm, which was well matched with the external diameter determined from TEM experiments (Fig. 2e). Additionally, two kinds of vertically equivalent reflections in (100) and (210) direction were observed. Taking into account two-dimensional tubular stacking, the two equidistance reflections with 8.4 nm in meridian were attributable as 3D hexagonally ordered tubular crystals as (101) and (211) reflections. On the basis of these results and measured densities, the number of molecules in tubular unit cell could be calculated as 3.1, suggesting that tubule **1** was based on the self-stacking of trimeric macrocycles. Subsequent optical spectra of **1** exhibited a blue-shifted absorption maximum and obvious fluorescence quenching in water compared with in ethanol, which revealed that trimeric macrocycles spontaneously stacked on each other to form well-ordered tubules through face-to-face arrangement (Supplementary Figs. 2 and 3). On the other hand, the absorption maximum of **2** was red-shifted in water accompanied with enhanced fluorescence intensity with respect to those observed in ethanol solution, indicating that the *H*-type aggregation of trimeric macrocycles transformed into fully overlapped *J*-type assemblies through side-by-side stacking due to the reduced length of alkyl chains (Supplementary Figs. 2 and 3). Notably, 2D XRD of **2** showed two kinds of vertically equivalent diffractions at equator and meridian (Fig. 2f). Considering one-dimensional stacking of tubules, the equidistance of 8.3 nm from equatorial diffractions could be indexed as intercolumnar distance, demonstrating the reduction of alkyl chains induced the inflation of tubules. Based on the increased outer diameter, the molecular number of **2** in one unit cell was estimated at 6.4, indicating that the inflation generated from self-assembly of expanded hexameric macrocycles. The unique regulation between hexameric and trimeric macrocycles by different length of terminal alkyl chains can be explained by mutual competition of π−π lateral stacking and phase separation from hydrophobic alkyl segments. Owing to the weak Van Der Waals interaction between butyl segments of **2**, the lateral strong π−π interaction rendered the bent-shaped aromatic molecules to aggregate into stable hexameric macrocycles with an overlapped side-by-side arrangement. However, the increment of alkyl length from butyl to decyl induced the strong hydrophobic interaction within aromatic apertures, leading the alkyl blocks to escape from hydrophilic oligoether segments. The special behavior drove the aromatic segments to rearrange into trimeric macrocycles with strong phase separation giving rise to the complete exposure of pyridines in the inner surface of aromatic walls.

### Different catalytic behavior

**Fig. 3:**
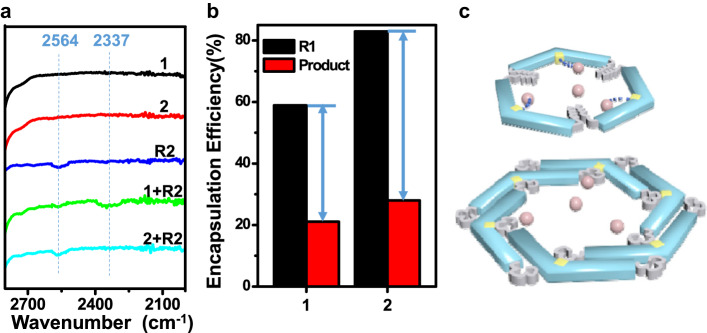
Mutual interaction between reagents and tubules. **a** FTIR spectra of **1**, **2**, R2, **1** with R2, **2** with R2. **b** Encapsulation efficiency of R1 and product by tubules **1** and **2**. **c** Schematic representation of interaction between R2 and pyridines in trimeric and hexameric macrocycles. Pink balls stand for R2.

Owing to the regular distribution of nitrogen on the tubular walls, both tubules with super-hydrophobic pores from the stacking of trimeric and hexameric macrocycles were well suitable to serve as heterogeneous catalysts for S_N_Ar reaction. Accordingly, comparable catalysis for nucleophilic substitution reaction of 1,3-dinitro-4-chlorobenzene (R1) and 1-octanethiol (R2) was performed with tubular catalysts **1** and **2** at ambient temperature, respectively. In spite of well-ordered porosity for both catalysts, a distinctly different catalytic behavior was observed. Interestingly, catalyst **1** with small aperture gave high catalytic activity up to 77% (Supplementary Fig. 5). However, although **2** possessed sufficient hydrophobic pores, the reaction did not proceed at all. To explore the disparate catalysis of **1** and **2**, the Fourier transform infrared (FTIR) experiments were further performed. Lower energy shift of S-H stretching band from 2564 cm^−1^ to 2337 cm^−1^ was clearly observed for catalyst **1** as a consequence of the interaction between S-H and exposed pyridine on the aromatic walls (Fig. 3a, Supplementary Fig. 6)^31^. Nonetheless, the S-H stretching vibration remained unchanged in the presence of catalyst **2**, indicating that there was no mutual effect between reagents and pyridine. The ineffective catalysis of **2** was attributable to the block of active pyridines by flexible butyl chains within hexameric aromatic walls (Fig. 3c). Remarkably, the blocked tubules exhibited preferable equilibrium uptake for hydrophobic reagent and product because of inflated aperture in contrast to **1** (Fig. 3b, Supplementary Fig. 7). Moreover, there was a greater difference in adsorption for reagent and product in presence of tubule **2** (Fig. 3b, Supplementary Fig. 7).

### Thermal response and efficient product turnover

**Fig. 4:**
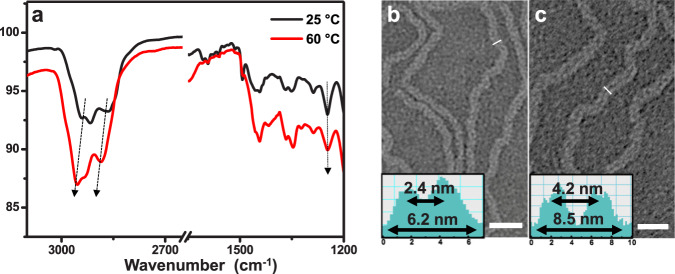
Tubular expansion upon heating. **a** FTIR spectra of **1** at 25 and 60 °C. Stained TEM images of **1** dried at 25 °C (**b**) and 60 °C (**c**), scale bar is 25 nm. Inset in (**b**) and (**c**) is corresponding density profile.

**Fig. 5:**
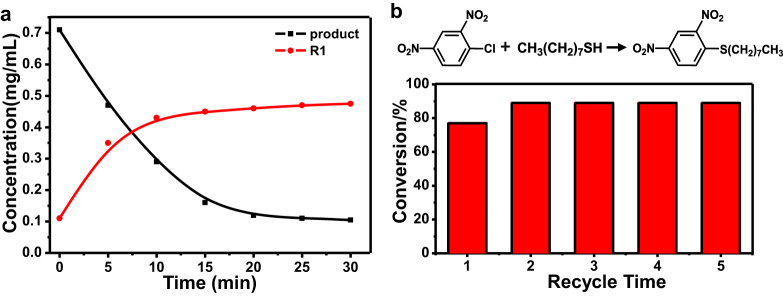
Exchange of product and R1 for S_N_Ar reaction. **a** Exchange of product and R1 within tubule **1** upon new addition of R1 at 60 °C after reaction. **b** Repeated cycles of conversion as a function of recycle times. The first recycle was without heating treatment.

The marked distinction in adsorption for reagent and product within expansive tubules with weak hydrophobic alkyl interaction motivated us to explore the thermo-responsive behavior of contracted tubule **1** through melting the decyl chains into molecular globules to reduce strong hydrophobic interaction. The melting behavior of decyl blocks was confirmed by temperature-dependent FTIR experiments (Fig. 4a)^32^. At ambient temperature, the film of **1** from aqueous solution showed two bands at 2918 cm^−1^ and 2863 cm^−1^, which corresponded to antisymmetric and symmetric CH_3_ and CH_2_ stretching vibration in the crystalline packing of ended alkyl chains. However, both bands shifted upward and gradually broadened upon heating above 60 °C, which implied the terminal alkyl chains tended to conformationally disorder. Meanwhile, the maximum adsorption was red-shifted from 320 to 340 nm accompanied with an increasing fluorescence intensity, suggesting that the *H*-type aggregations changed into fully overlapped *J*-type assemblies (Supplementary Fig. 8). To confirm this rearranged aggregation upon heating, TEM and XRD experiments were subsequently performed. Upon heating to 60 °C, TEM micrographs of **1** from negatively stained samples generated an expansion with external and inner diameter of 8.5 and 4.2 nm proven by density profile taken perpendicular to the long axis of the tubules (Fig. 4b, c). The expansion was further examined by XRD reflection. The membrane of **1** from slow evaporation of aqueous solution at 60 °C displayed three reflections with the ratio of 1:$\sqrt 3$:2 at the small-angle range, which could be assigned as 2D hexagonal columnar structures with corresponding lattice parameter of 7.0 nm (Supplementary Fig. 9). Based on the increased column lattice upon heating, the molecular number of **1** in one unit cell was estimated at 6.3 at 60 °C, indicating that the inflation generated from self-assembly of expanded hexameric macrocycles. Given these results, we concluded that the dynamic tubules based on trimeric macrocycles could readily rearrange into hexameric macrocycles that stacked into inflated tubules through conformational change of terminal alkyl chains upon heating, which was observed fully reversible on next heating−cooling cycles. Notably, the expansion by thermo triggers facilitated the encapsulation of reagents. Supplementary Fig. 10a showed that the encapsulated R1 was increased by 30% within tubules **1** upon heating due to the expansive aperture. When cooling to room temperature, the inflated tubules were contracted with concentrated reagents to generate a preferable catalytic conversion up to 89% (Supplementary Fig. 10b). Importantly, after completion of the reaction, subsequent heating resulted in the exchange of products and reagents (Fig. 5a, Supplementary Fig. 11). Upon addition of new reactants, the reagents were gradually encapsulated in tubular catalyst while the products were spontaneously released, which was confirmed by high performance liquid chromatography (HPLC) with time variation. The escaped products were readily obtained by ultrafiltration, and thus subsequent catalysis could be continued through the contraction of tubular catalyst on cooling. The initial catalytic ability was completely maintained during five cycles of pulsating motion and the tubular structure kept unchanged (Fig. 5b, Supplementary Fig. 12). From above results, we concluded that the tubular catalyst **1** exhibited outstanding catalytic performance in terms of high efficiency, recyclability and reusability. Moreover, we have additionally performed nucleophilic substitutions with different substrates catalyzed by tubule **1** and gained great catalytic performance, which suggested the tubular catalyst was applicable to S_N_Ar reaction for various thiols (Supplementary Fig. 13). Notably, compared with general porous heterogeneous catalysts, the supramolecular catalyst was additionally endowed with dynamic pumping by thermo triggers, which contributed greatly to the effective exchange of products and reagents for continuous catalysis.

In summary, a responsive tubule with dynamic pulsating was fabricated through self-stacking of trimeric macrocycles, which could readily inflate into hexameric macrocycles by thermo triggers. Owing to the periodical distribution of nitrogen on the inner surface of trimeric macrocycles, the contracted tubules were employed as efficient heterogeneous catalyst for S_N_Ar reaction with high catalytic activities. Nevertheless, the catalyst could be suspended immediately upon heating due to the block of active sites. The inactivation of transient state at high temperature was remarkably different from conventional catalysts that accelerated reaction upon heating^33,34^. Importantly, the inflation of tubules facilitated the exchange of products and reagents due to the increased entropy in inflated pores. Such strategy of dynamic catalytic device with regulable pore for exchange of products and reagents would provide a new insight into continuous product turnover.

## Methods

### Materials, general and synthesis of compounds 1 and 2

See the Supplementary Methods.

### TEM experiments

To investigate the structures of **1** and **2** in aqueous solution (0.01 wt%), a drop of solution was placed on a graphene-supported film and the solution was allowed to evaporate under ambient conditions. The dried specimen was observed by using a JEM ARM 200F machine operated at 80 kV to determine aromatic wall within tubules. The wall was further imaged using probe aberration corrector by STEM model to observe pore size. The membranes were transferred onto carbon-coated copper grid and then stained by depositing a drop of uranyl acetate aqueous solution (0.1 wt%) onto the surface of the loaded grid. When dry, the out-diameter of tubules was detected by JEM ARM 200F machine at 80 kV.

### General procedure for S_N_Ar reaction

To the aqueous solution of catalyst **1** (or **2**) (0.0028 mmol), 1,3-dinitro-4-chlorobenzene (0.014 mmol) and 1-octanethiol (0.016 mmol) were added. The reaction mixture was stirred for 2 h at room temperature in aerobic environment. After the completion of the reaction, the reaction solution was evaporated and acetonitrile was added. The mixture of acetonitrile solution was subsequently injected into the HPLC. The conversion was confirmed by analytical-C18 column (mobile phase: acetonitrile, flow rate: 1.0 mL min^−1^, *λ* = 254 nm). The qualitative analysis was determined by the retention time compared with the pure product purchased from commercial suppliers or after purification and characterization, which is as following: *t*_R_ = 3.2 min (R1); *t*_R_ = 5.4 min (product).

### FTIR experiments

To the aqueous solution of tubule **1** (or **2**) (3 wt%), 1-octanethiol (a mole ratio R2/**1** or **2** of 1:5) was added and sonicated for 30 min. The mixture was further cast on spectrometer using ATR mode.

### Adsorption of R1, 4-methoxybenzenethiol and product

The adsorption of R1, 4-methoxybenzenethiol (R3) or product was performed by mixing tubules **1** (or **2**) (1.0 mg) with R1 (0.6 mg), R3 (0.4 mg) or product (1.0 mg) solution and the mixture sample was sonicated for 30 min at room temperature. Then, the mixed solution was filtered through an ultrafiltration spin column. The residual concentration of the adsorbate in each filtrate was determined by UV−vis spectroscopy under 236 nm (R1), 238 nm (R3) and 332 nm (product). The encapsulation efficiency (in %) by the tubules was determined by the following equation:

1 $${{{\mathrm{Encapsulation}}}}\,{{{\mathrm{efficiency}}}} = \frac{{C_{{{\mathrm{o}}}} - C_{{{\mathrm{e}}}}}}{{C_{{{\mathrm{o}}}}}} \times 100,$$

where *C*_o_ (mmol^−1^) and *C*_e_ (mmol^−1^) are the initial and equilibrium concentration of the adsorbates, respectively.

### S_N_Ar reaction in heating−cooling recycle

To the aqueous solution of catalyst **1** (or **2**) (0.0028 mmol), 1,3-dinitro-4-chlorobenzene (0.014 mmol) and 1-octanethiol (0.016 mmol) were added at 60 °C. Upon cooling, the reaction was continued following the above-mentioned procedure.

### Exchange of products and reagents with catalyst 1

After completion of the S_N_Ar reaction, the reaction mixture was loaded into dialysis bag and socked with the solution containing 1,3-dinitro-4-chlorobenzene (0.014 mmol) and 1-octanethiol (0.016 mmol) under stirring at 60 °C. Twenty microliters of solution in dialysis bag was taken out at predetermined time intervals and acetonitrile was added after evaporation. The acetonitrile solution was injected into HPLC to determine the adsorbates.

### Supplementary information

Supplementary Information

## Data Availability

The authors declare that the data supporting the findings of this study are available within the article and Supplementary Information, or from the corresponding author upon reasonable request.

## References

[1] Traut, T. *Allosteric Regulatory Enzymes* (Springer, New York, 2008).

[2] Dong Z, Luo Q, Liu J (2012). Artificial enzymes based on supramolecular scaffolds. Chem. Soc. Rev..

[3] Blanco V, Leigh DA, Marcos V (2015). Artificial switchable catalysts. Chem. Soc. Rev..

[4] Wiester MJ, Ulmann PA, Mirkin CA (2011). Enzyme mimics based upon supramolecular coordination chemistry. Angew. Chem. Int. Ed..

[5] Lubitz W, Reijerse E, Gastel Mvan (2007). [NiFe] and [FeFe] hydrogenases studied by advanced magnetic resonance techniques. Chem. Rev..

[6] Weingarten AS (2014). Self-assembling hydrogel scaffolds for photocatalytic hydrogen production. Nat. Chem..

[7] Koblenz TS, Wassenaar J, Reek JNH (2008). Reactivity within a confined self-assembled nanospace. Chem. Soc. Rev..

[8] Küchler A, Yoshimoto M, Luginbühl S, Mavelli F, Walde P (2016). Enzymatic reactions in confined environments. Nat. Nanotech..

[9] Wang Q-Q (2016). Self-assembled nanospheres with multiple endohedral binding sites pre-organize catalysts and substrates for highly efficient reactions. Nat. Chem..

[10] Meeuwissen J, Reek JNH (2010). Supramolecular catalysis beyond enzyme mimics. Nat. Chem..

[11] Taniguchi A, Shimizu Y, Oisaki K, Sohma Y, Kanai M (2016). Switchable photooxygenation catalysts that sense higher-order amyloid structures. Nat. Chem..

[12] Yoon HJ, Kuwabara J, Kim J-H, Mirkin CA (2010). Allosteric supramolecular triple-layer catalysts. Science.

[13] Zhang, Q., Wang, W.-Z., Yu, J.-J., Qu, D.-H. & Tian, H. Dynamic self-assembly encodes a tri-stable Au–TiO_2_ photocatalyst. *Adv. Mater*. 10.1002/adma.201604948 (2017). 10.1002/adma.201604948 27874232

[14] Rodríguez-Llansola F, Escuder B, Miravet JF (2009). Switchable perfomance of an L-proline-derived basic catalyst controlled by supramolecular gelation. J. Am. Chem. Soc..

[15] Wei Y, Han S, Kim J, Soh S, Grzybowski BA (2010). Photoswitchable catalysis mediated by dynamic aggregation of nanoparticles. J. Am. Chem. Soc..

[16] Qin L (2013). Supramolecular assemblies of amphiphilic L-Proline regulated by compressed CO_2_ as a recyclable organocatalyst for the asymmetric aldol reaction. Angew. Chem. Int. Ed..

[17] Huang Z (2012). Pulsating tubules from noncovalent macrocycles. Science.

[18] Yu S, Azzam T, Rouiller I, Eisenberg A (2009). “Breathing” vesicles. J. Am. Chem. Soc..

[19] Vriezema DM (2005). Self-assembled nanoreactors. Chem. Rev..

[20] Percec V (2004). Self-assembly of amphiphilic dendritic dipeptides into helical pores. Nature.

[21] Jin W (2005). Self-assembled graphitic nanotubes with one-handed helical arrays of a chiral amphiphilic molecular grapheme. Proc. Natl Acad. Sci. USA.

[22] Wang C (2008). Controlled self-assembly manipulated by charge-transfer interactions: from tubes to vesicles. Angew. Chem. Int. Ed..

[23] Chen L-J (2014). Smart stimuli-responsive spherical nanostructures constructed from supramolecular metallodendrimers via hierarchical self-assembly. J. Am. Chem. Soc..

[24] Xie, S. et al. Intelligent mesoporous materials for selective adsorption and mechanical release of organic pollutants from water. *Adv. Mater*. 10.1002/adma.201800683 (2018). 10.1002/adma.201800683 29782684

[25] Kawauchi T (2010). Separation of C_70_ over C_60_ and selective extraction and resolution of higher fullerenes by syndiotactic helical poly(methyl methacrylate). J. Am. Chem. Soc..

[26] Wang H, Wang Y, Shen B, Liu X, Lee M (2019). Substrate-driven transient self-assembly and spontaneous disassembly directed by chemical reaction with product release. J. Am. Chem. Soc..

[27] Tang J (2015). Synthesis of nitrogen-doped mesoporous carbon spheres with extra-large pores through assembly of diblock copolymer micelles. Angew. Chem. Int. Ed..

[28] He L, Weniger F, Neumann H, Beller M (2016). Synthesis, characterization, and application of metal nanoparticles supported on nitrogen-doped carbon: catalysis beyond electrochemistry. Angew. Chem. Int. Ed..

[29] Arrigo R (2015). Nature of the N−Pd interaction in nitrogen-doped carbon nanotube catalysts. ACS Catal..

[30] Wu S (2017). Supramolecular nanotubules as a catalytic regulator for palladium cations: applications in selective catalysis. Angew. Chem. Int. Ed..

[31] Baranowska K, Piwowarska N, Herman A, Dołęga A (2012). Imidazolium silanethiolates relevant to the active site of cysteine proteases. A cooperative effect in a chain of NH^+^S^−^ hydrogen bonds. N. J. Chem..

[32] Li W, Yi S, Wu Y, Wu L (2006). Thermotropic mesomorphic behavior of surfactant-encapsulated polyoxometalate hybrids. J. Phys. Chem. B.

[33] Dai W (2016). Lewis acid catalysis confined in zeolite cages as a strategy for sustainable heterogeneous hydration of epoxides. ACS Catal..

[34] Yang X (2019). Quantification of active sites and elucidation of the reaction mechanism of the electrochemical nitrogen reduction reaction on vanadium nitride. Angew. Chem. Int. Ed..

