# Author Correction: Catalytically-active porous assembly with dynamic pulsating motion for efficient exchange of products and reagents

**DOI:** 10.1038/s42004-023-01078-y

**Published:** 2023-12-13

**Authors:** Shanshan Wu, Liping Huang, Yu Hou, Xin Liu, Jehan Kim, Yongri Liang, Jiong Zhao, Liwei Zhang, Hongbing Ji, Myongsoo Lee, Zhegang Huang

**Affiliations:** 1 Fine Chemical Industry Research Institute and PCFM Lab, School of Chemistry, Sun Yat-sen University, Guangzhou, 510275 PR China; 2 State Key Laboratory for Supramolecular Structure and Materials, College of Chemistry, Jilin University, Changchun, 130012 PR China; 3 Pohang Accelerator Laboratory, Postech, Pohang, Gyeongbuk Korea; 4 College of Materials Science and Engineering, Beijing Institute of Petrochemical Technology, Beijing, 102617 PR China; 5 Department of Applied Physics, The Hong Kong Polytechnic University, Hung Hom, Kowloon, Hong Kong

**Keywords:** Porous materials, Self-assembly

Correction to: *Communications Chemistry* 10.1038/s42004-020-0259-4, published online 24 January 2020.

In this article the funding from ‘the Natural Science Foundation of Guangdong Province for Distinguished Young Scholars (2019B151502051)’ was omitted from acknowledgement. The original article has been corrected.

