# Supplementary material for: Catalytically-active porous assembly with dynamic pulsating motion for efficient exchange of products and reagents
Source: Commun Chem. 2020 Jan 24;3:11. doi: 10.1038/s42004-020-0259-4 (PMC9814577; doi:10.1038/s42004-020-0259-4)
Supplement: Supplementary file 1 — Supplementary Information [file 42004_2020_259_MOESM1_ESM.pdf]

Supplementary Information

1. Supplementary Methods

Materials

2,6-Dibromo-4-hydroxypyridine, 4,4'-dibromobiphenyl, and iodine monochloride (1.0 M solution in dichloromethane) from Aldrich, ethyl L-(-)-lactate, tetrakis (triphenylphosphine) palladium (0) (99 %) from TCI, and 3-chloro-2-chloromethyl-1-propene (96 %) from Acros were used as received. Alkyl substituted phenyl boronic acids were synthesized according to previously reported method[1]. Unless otherwise indicated, all starting materials were obtained from commercial suppliers and were used without purification. Tetrahydrofuran (THF) was dried by distillation from sodium–benzophenone immediately prior to use. Dichloromethane (DCM) was dried by distillation from CaH_2. Distilled water was polished by ion exchange and filtration. Visualization was accomplished with UV light, iodine vapor. Flash chromatography was carried out with Silica Gel 60 (230-400 mesh) from EM Science.

General

The purity of the products was checked by thin-layer chromatography (TLC; Merck, silica gel 60). High performance liquid chromatography (HPLC) was performed for further purification by using Hanban Sci&Tech NP7000 SERIALS PUMP, NU3000 SERIALS UV/VIS DETECOR, C18 column (Hedera ODS-2 column). ^{1}H-NMR and ^{13}C-NMR spectra were recorded from $CDCl_3$ or d_6-DMSO solutions on Bruker AVANCE III 400MHz. All compounds are subjected to ^{1}H NMR analysis to confirm ⩾98% sample purity. Chemical shifts were reported in ppm relative to the residual solvent peak ($CDCl_3$: ^{1}H, 7.28; ^{13}C, 77.23). Multiplicity was indicated as follows: s (singlet), d (doublet), t (triplet), and m (multiplet). Coupling constants are reported in Hz. MALDI TOF-MS spectroscopy (MALDI-TOF-MS) was performed on a Bruker ESI-Q-TOF maXis 4G using α-cyano-4-hydroxy cinnamic acid (CHCA) as a matrix. The Uv/vis spectra were obtained from a Metash UV-8000S Spectrophotometer. The fluorescence spectra were obtained from a

Hitachi F-4500 Fluorescence Spectrophotometer. The two-dimensional X-ray scattering experiments were carried out at the 3C beamline in Pohang Accelerator Laboratory. The transmission electron microscopy (TEM) was performed at 80 kV using JEM ARM 200 machine. The acquisition time for bright field (BF) imaging was 1 s using the smallest objective lens aperture. Annular dark field (ADF)-STEM imaging was also conducted using JEM ARM 200F operated at 80 kV with a probe aberration corrector. Annular dark field (ADF) images were acquired at a 20 mrad convergence angle, collection angle from 50 mrad to 180 mrad and acquisition time 40 μs per pixel. Fourier transform infrared (FTIR) experiments were carried out on the Agilent 670-IR spectrometer using ATR mode under ambient conditions.

Synthesis of compounds 1 and 2

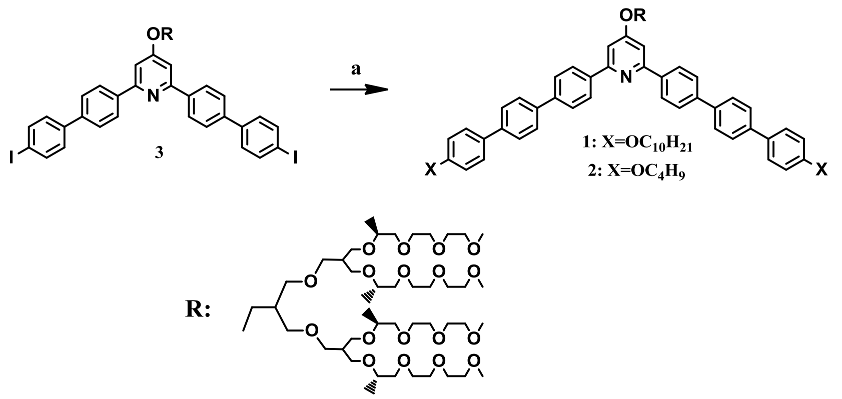

Synthesis of compound 1: Compound 3 (0.50 g, 0.32 mmol), and 4-(n-decyloxy)-phenylboronic acid (0.18 g, 0.65 mmol) were dissolved in degassed THF (30 mL). Degassed K_2CO_3 aqueous (2M, 30 mL) was added to the above mixture and then $Pd(PPh_3)_4$ (0.06 g, 0.05 mmol) was added carefully. The mixture was refluxed for 24 hours with vigorous stirring under argon atmosphere. After completion of the reaction as monitored by TLC, the reaction mixture was cooled down to room temperature and extracted with ethyl acetate three times. The combined organic layer was dried over anhydrous $MgSO_4$. The filtrate was condensed under reduced pressure and purified by silica gel flash column using ethyl acetate/methanol (10:1, v/v) as eluent. Finally the purified product was subjected to further purification by HPLC to yield 0.40 g (71 %) of a colorless oil.

Compound 1. ^{1}H-NMR (400 MHz, $CDCl_3$, δ): 8.28 (d, J = 8.0 Hz, 4H), 7.81 (d, J = 8.0 Hz, 8H), 7.70 (d, J = 8.0 Hz, 4H), 7.62(d, J = 8.0 Hz, 4H), 7.33 (s, 2H), 7.03 (d, J = 8.0 Hz, 4H), 4.29 (d, J

= 8.0 Hz, 2H), 4.06 (t, *J* = 8.0 Hz, 4H), 3.64-3.37 (m, 72H), 2.48 (m, 1H), 2.16-2.12 (m, 2H), 1.88 (m, 4H), 1.53 (m, 4H), 1.35 (m, 24H), 1.12 (d, *J* = 8 Hz, 12H), 0.92 (t, *J* = 4.0 Hz, 8Hz, 6H). ^{13}C-NMR (300 MHz $CDCl_3$, δ), 158.97, 158.21, 141.39, 140.15, 138.96, 138.57, 133.04, 128.09, 127.59, 127.47, 127.21, 127.16, 114.97, 77.41, 77.29, 77.09, 76.77, 75.14, 74.92, 72.02, 70.83, 70.69, 70.59, 68.23, 67.49, 59.07, 40.91, 31.98, 29.67, 29.64, 29.49, 26.40, 26.15, 22.75, 17.18, 17.17, 14.18. MALDI-TOF mass: m/z calcd. for **1** $[M+H]^+$, 1770.36, $[M+Na]^+$, 1792.35; found: $[M+H]^+$, 1770.12, $[M+Na]^+$, 1791.99.

Synthesis of compound 2: Compound 3 (0.50 g, 0.32 mmol), and 4-(n-butyloxy)-phenylboronic acid (0.13 g, 0.65 mmol) were dissolved in degassed THF (30 mL). Degassed K_2CO_3 aqueous (2M, 30 mL) was added to the above mixture and then $Pd(PPh_3)_4$ (0.06 g, 0.05 mmol) was added carefully. The mixture was refluxed for 24 hours with vigorous stirring under argon atmosphere. After completion of the reaction as monitored by TLC, the reaction mixture was cooled down to room temperature and extracted with ethyl acetate three times. The combined organic layer was dried over anhydrous $MgSO_4$. The filtrate was condensed under reduced pressure and purified by silica gel flash column using ethyl acetate/methanol (10:1, v/v) as eluent. Finally the purified product was subjected to further purification by HPLC to yield 0.38 g (74 %) of a white solid.

Compound 2. ^{1}H-NMR (400 MHz, $CDCl_3$, ppm): δ 8.28 (d, *J* = 8.0 Hz, 4H), 7.81 (d, *J* = 8.0 Hz, 8H), 7.70 (d, *J* = 8.0 Hz, 4H), 7.62(d, *J* = 8.0 Hz, 4H), 7.33 (s, 2H), 7.03 (d, *J* = 8.0 Hz, 4H), 4.29 (d, *J* = 8.0 Hz, 2H), 4.06 (t, *J* = 8.0 Hz, 4H), 3.64-3.37 (m, 72H), 2.48 (m, 1H), 2.16-2.12 (m, 2H), 1.88 (m, 4H), 1.53 (m, 4H), 1.12 (d, *J* = 8 Hz, 12H), 0.92 (t, *J* = 4.0 Hz, 8Hz, 6H). ^{13}C-NMR (300 MHz), 158.82, 157.88, 141.28, 139.87, 138.70, 138.24, 132.83, 127.95, 127.44, 127.32, 127.01, 114.79, 77.27, 76.96, 76.64, 74.98, 74.76, 71.86, 70.67, 70.54, 70.45, 67.73, 58.94, 40.83, 39.88, 31.29, 19.21, 17.03, 13.80. MALDI-TOF mass: m/z calcd. for **1** $[M+H]^+$, 1602.05, $[M+Na]^+$, 1624.04; found: $[M+H]^+$, 1602.50, $[M+Na]^+$, 1624.42.

2. Supplementary Figures

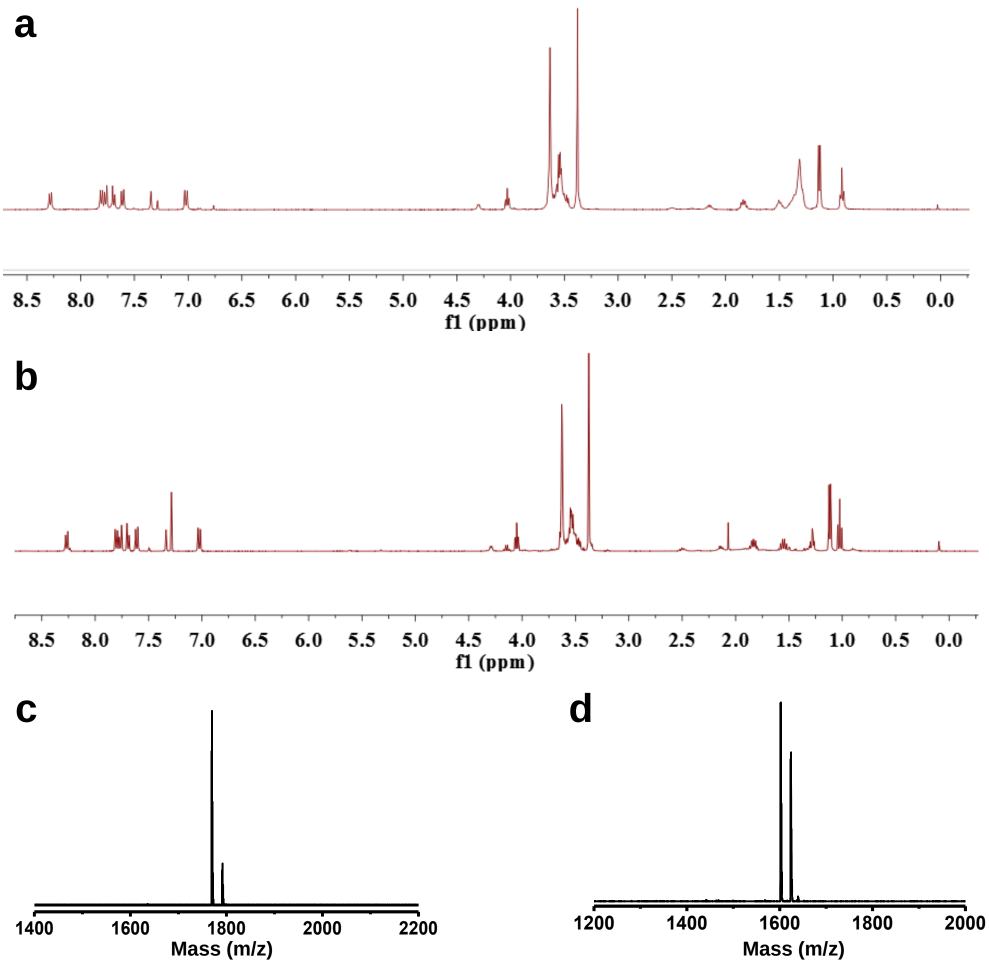

Supplementary Figure 1 Characterization of molecular structures. ^{1}H-NMR spectra of **1** (**a**), **2** (**b**); MALDI-TOF mass spectra of **1** (**c**), **2** (**d**).

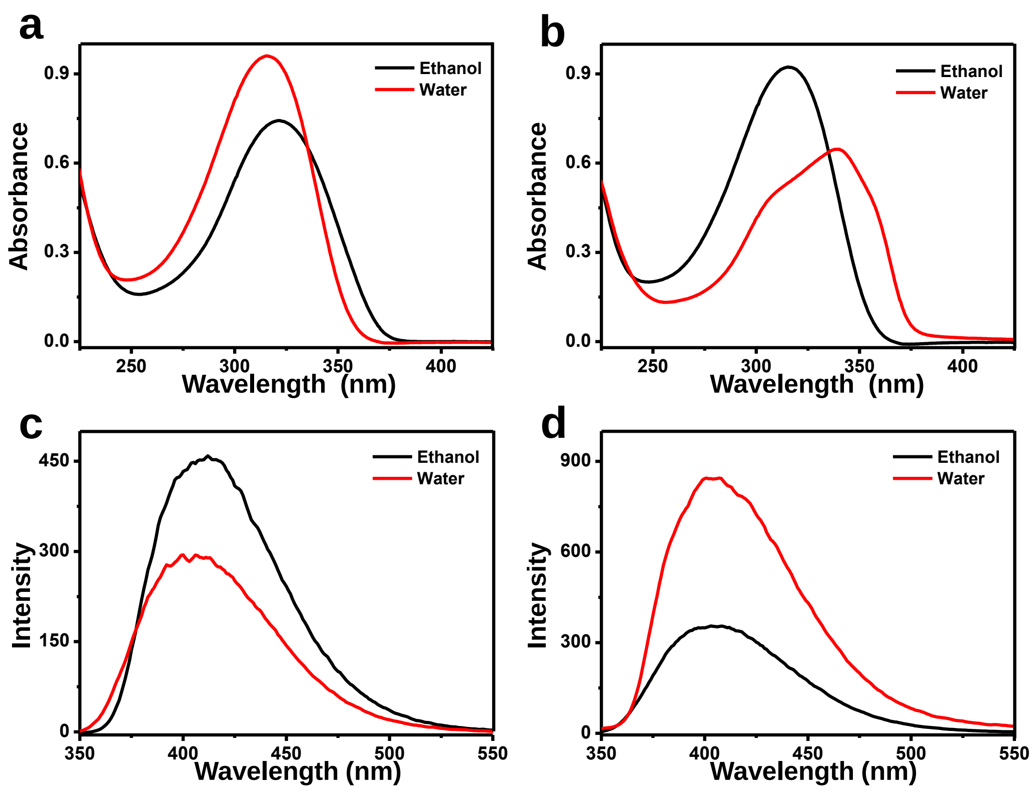

Supplementary Figure 2 Optical properties of **1** and **2** and their aggregations. The absorption spectra (**a**) and the fluorescence spectra (**c**) of **1** in ethanol and water solution (0.01 wt%); The absorption spectra (**b**) and the fluorescence spectra (**d**) of **2** in ethanol and water solution (0.01 wt %).

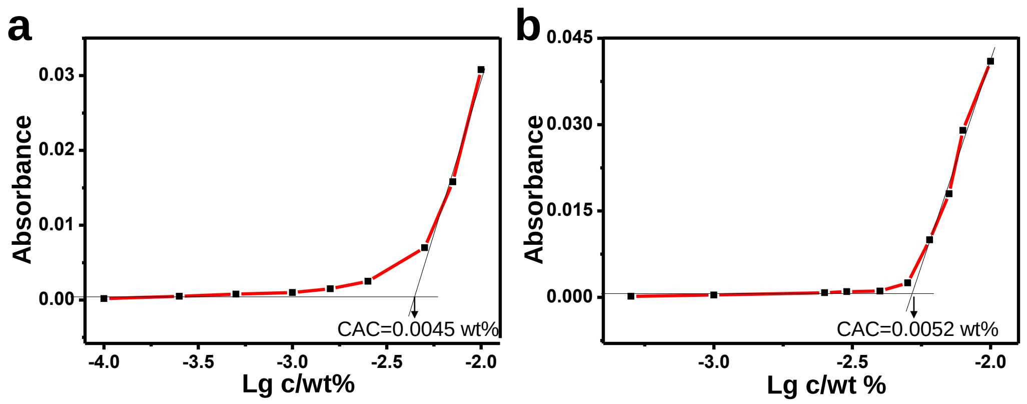

Supplementary Figure 3 The critical aggregation concentration of **1** and **2**. Relationship of the absorbance and the concentration of **1** (**a**) and **2** (**b**) in aqueous solution. (λ= 400 nm).

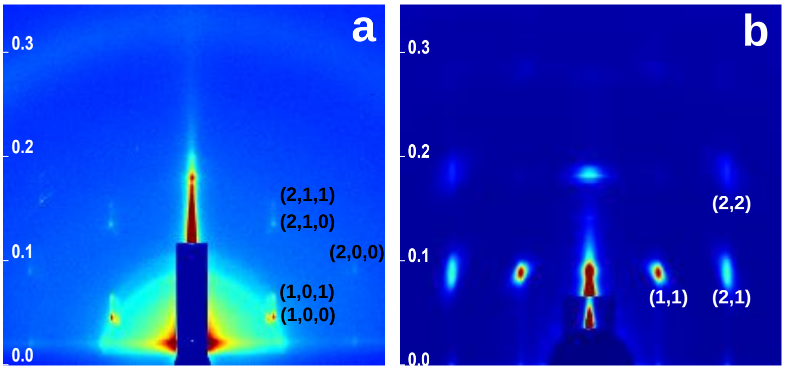

Supplementary Figure 4 2D XRD patterns of **1** (**a**) and **2** (**b**).

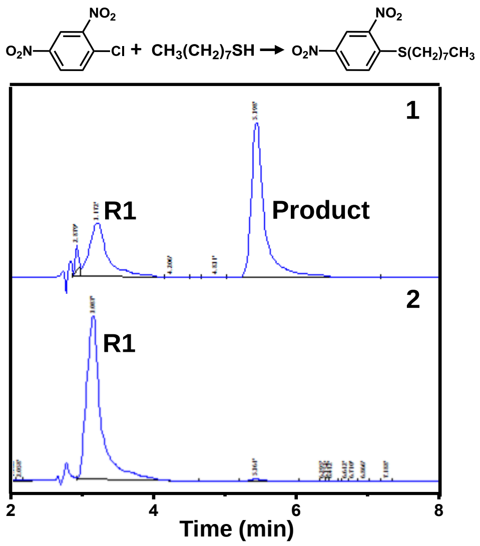

Supplementary Figure 5 The HPLC data for S_NAr reaction catalyzed by tubular catalyst **1** and **2**.

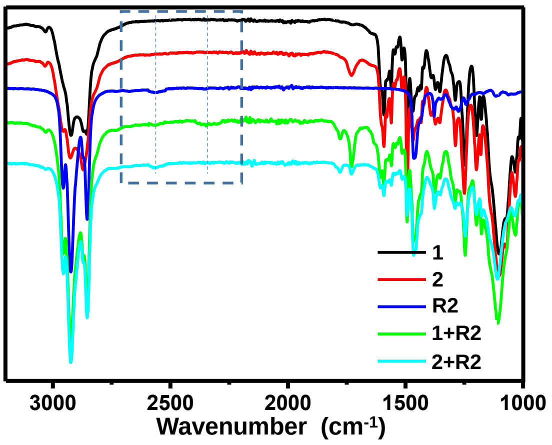

Supplementary Figure 6 Full view for FTIR spectra of **1**, **2**, R2, **1** with R2, **2** with R2.

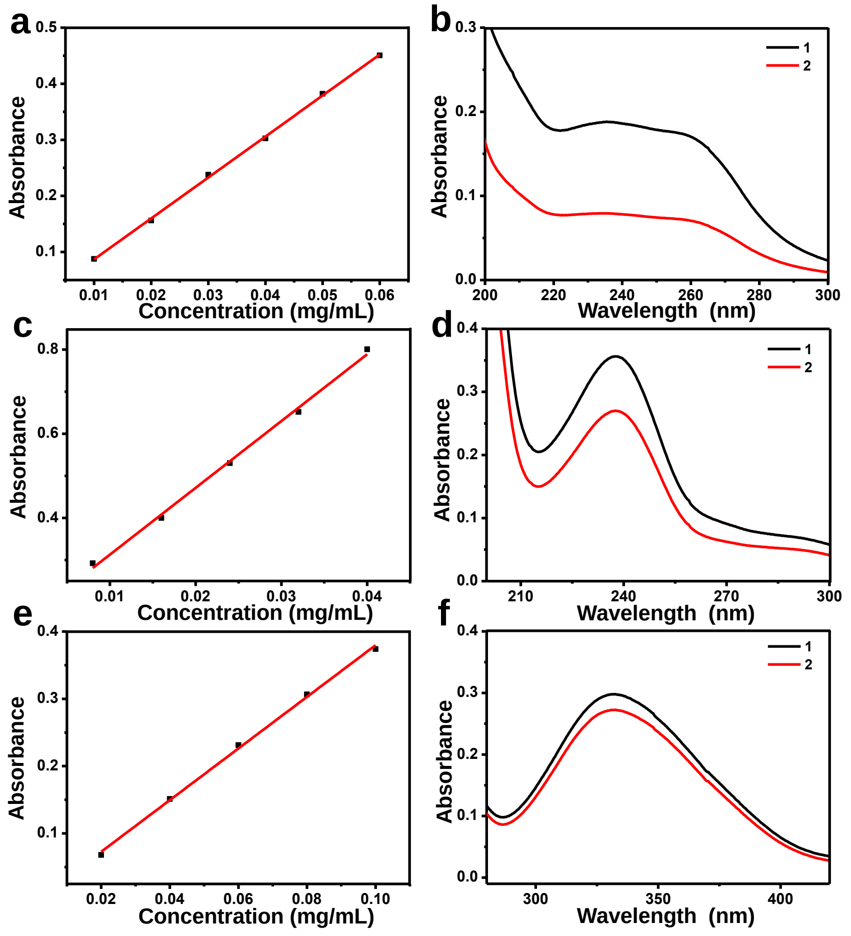

Supplementary Figure 7 Optical properties of R1, R3 and product. Standard curve for R1 (**a**): Abs=0.0137+7.3057*c (λ=236 nm), R=0.9992, R3 (**c**): Abs=0.154+15.875*c (λ=238 nm), R=0.9962, and product (**e**): Abs=-0.0039+3.8355*c (λ=332 nm), R=0.9980. The residual R1 (**b**), R3 (**d**) and product (**f**) after adsorption by tubule **1** and **2**. The adsorption efficiency for R3 was 68.2 % by tubule **1** and 81.7 % by tubule **2**.

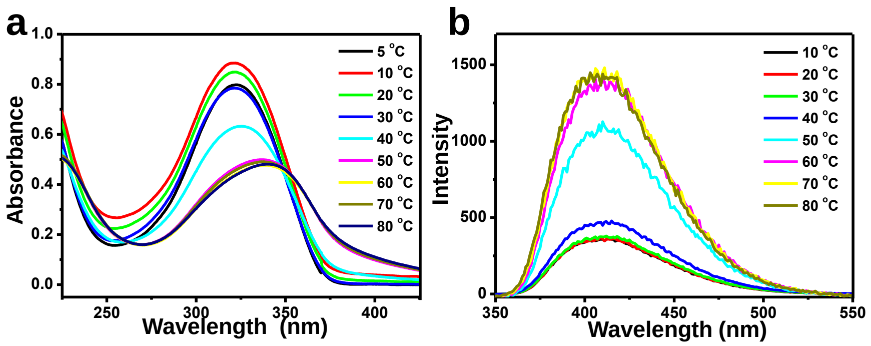

Supplementary Figure 8 Thermo-responsive optical properties of **1**. The absorption spectra (**a**) and the fluorescence spectra (**b**) of **1** in aqueous solution (0.01 wt%) with temperature variation.

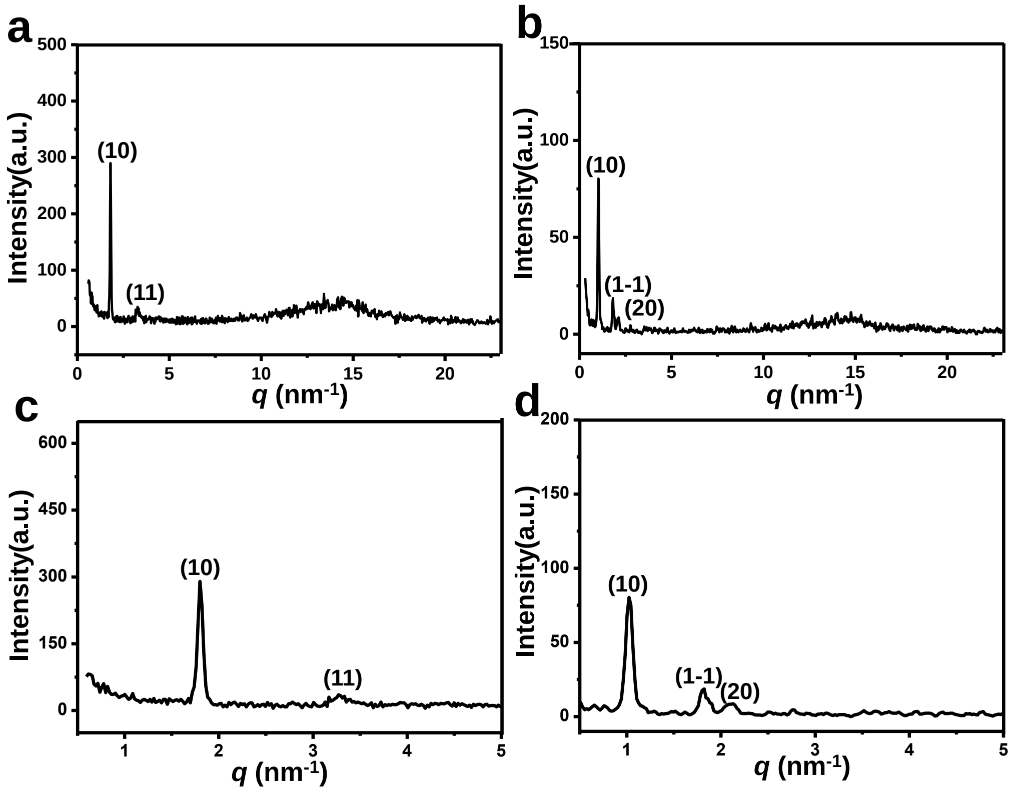

Supplementary Figure 9 XRD results of **1** at different state. Full view for XRD pattern of **1** dried at 25 °C (**a**) and 60 °C (**b**); Small-angle XRD pattern of **1** dried at 25 °C (**c**) and 60 °C (**d**). **1** dried at 25 °C and 60 °C displayed several reflections in the small angle and a diffuse diffraction peak in the wide-angle region, indicative of the formation of 2D liquid crystals with an intermolecular distance of 4.3 Å. Taking into account two-dimensional tubular stacking, two sharp small-angle reflections of **1** at 25 °C could be indexed as a 2D oblique columnar structure with a lattice constant of 4.7 nm and characteristic angle of 50°. The reflections of **1** at 60 °C in small angle could be assigned as a 2D hexagonal column with a lattice parameter of 7.0 nm. On the basis of these results and measured densities, the number of molecules of **1** at 25 °C and 60 °C in one tubular unit cell could be calculated as 2.8 and 6.3. ($N = a^2 d \sin(\gamma) N_A \rho / M$, d represents intermolecular distance while ρ stands for the density of the liquid crystals.)

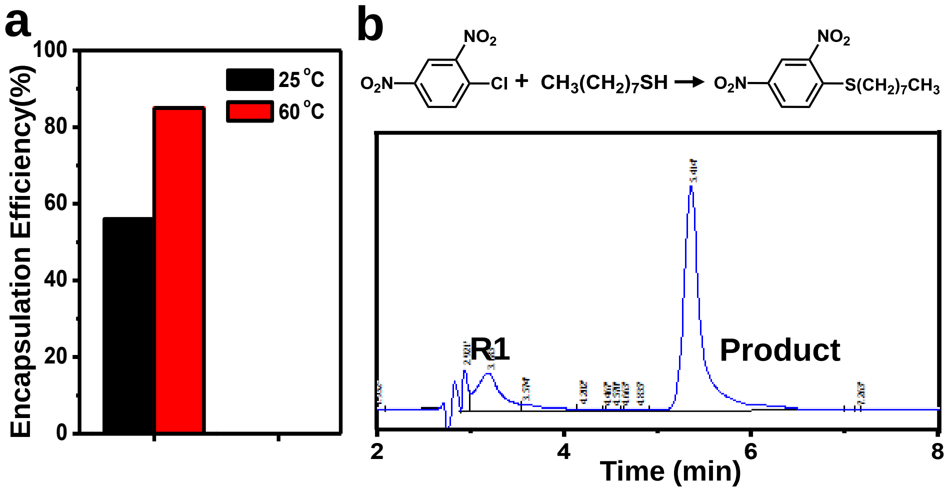

Supplementary Figure 10 (**a**) Encapsulation efficiency of R1 by tubule **1** at 25 °C and 60 °C; (**b**) The HPLC data for S_NAr reaction catalyzed by tubular catalyst **1** with heating treatment.

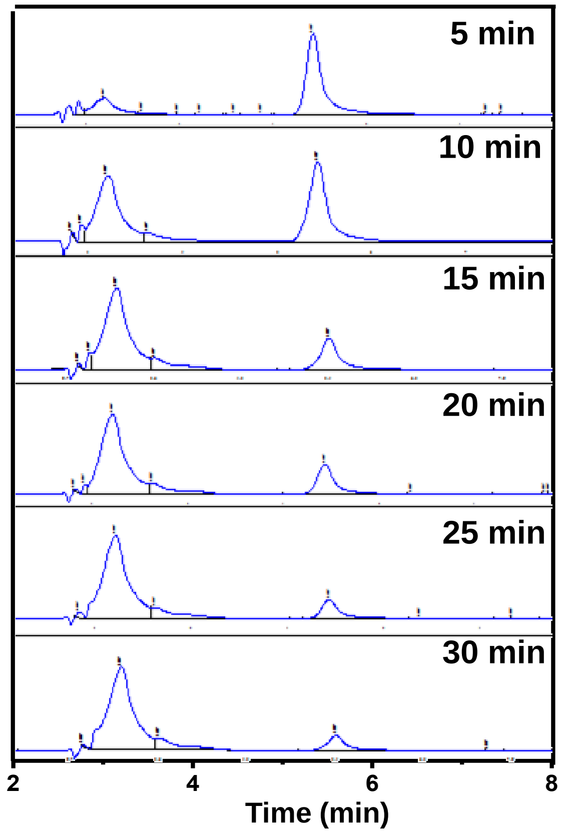

Supplementary Figure 11 The raw HPLC data for exchange of product and R1 within tubule **1** upon new addition of R1 at 60 °C after reaction.

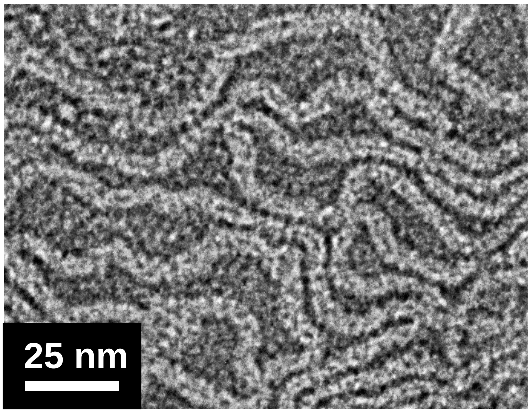

Supplementary Figure 12 The tubular structure of catalyst **1** after five cycles of catalysis.

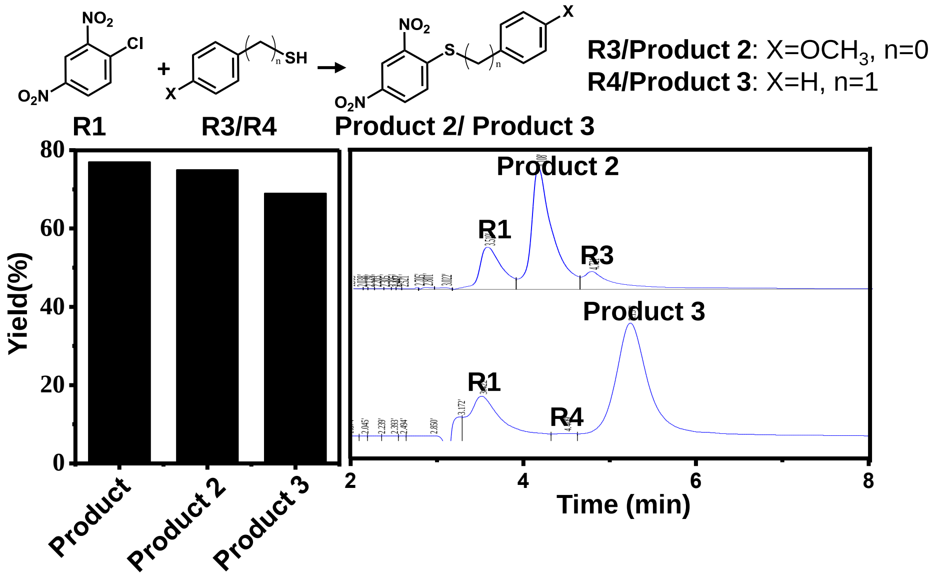

Supplementary Figure 13 The HPLC data for S_NAr reaction between R1 and 4-methoxybenzenethiol (R3)/ benzyl mercaptan (R4) catalyzed by tubular catalyst **1** without heating treatment. The yield for product 2/product 3 was determined as 75% and 69%.

3. Supplementary Reference

1. Wu, S., et al. Supramolecular nanotubules as a catalytic regulator for palladium cations: applications in selective catalysis. *Angew. Chem. Int. Ed.* **56**, 11511-11514 (2017).
